# Advances in anterior segment examination

**Published:** 2019-12-17

**Authors:** Ritika Mukhija, Noopur Gupta

**Affiliations:** 1 Senior Resident: Dr Rajendra Prasad Centre for Ophthalmic Sciences, AIIMS, New Delhi, India.; 2 Associate Professor of Ophthalmology, Dr Rajendra Prasad Centre for Ophthalmic Sciences, AIIMS, New Delhi, India.

**Corneal imaging techniques are used to assess the structure and function of the cornea and anterior segment. They are crucial for diagnosing and treating a wide variety of ocular diseases.**

Corneal and ocular surface imaging is an ever-advancing field in ophthalmology. There have been several innovations in imaging technologies, such as rotating Scheimpflug, anterior segment optical coherence tomography (ASOCT) and confocal microscopy. Investigative technologies like ocular surface analysers have helped to understand and manage anterior segment diseases in newer ways. In this article, we discuss various techniques, their advantages, and their limitations.

## Corneal topography and tomography

The growing popularity of refractive surgeries has prompted rapid advancements in corneal imaging. Corneal topography helps to map the shape and features of the corneal surface. Placido's disc-based and slit-scanning system are two common technologies in use today. Tomographers, generate 3D images of the anterior segment of a cornea which gives information about its thickness. Scheimpflug imaging and optical coherence tomography (OCT), are two examples of tomography.^1^

## Placido disc-based keratoscopy

**Figure 1:**
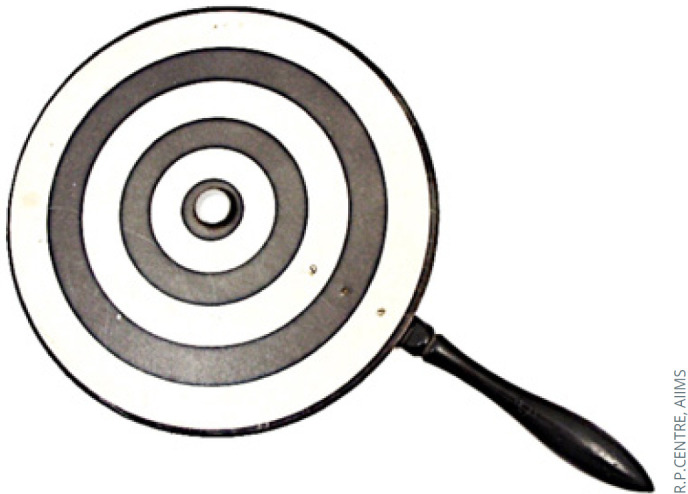
Placido disk with alternate light and dark concentric rings

Placido's disc-based videokeratoscopy (Figure 1) is a common and easy-to-perform topography technique to study the anterior corneal surface. It provides information on cornea's shape (central power, simulated keratometry, corneal asphericity) and aberrometry. It is useful in the diagnosis of corneal ectatic disorders like keratoconus and while fitting contact lenses. It also helps in intraocular lens power calculation for cataract surgery in patients with irregular corneas, follow-up, and management of post-keratoplasty (corneal transplantation) astigmatism and dry eye assessment (with non-invasive tear break up time).

A limitation with this technique is that it covers a limited corneal surface area (about 60 per cent). It does not provide information about the posterior corneal surface, which is important in the early diagnosis of ectasia.

## Slit-scanning

Slit-scanning elevation topography combines projection of a slit of light with Placido's disc keratoscopy to get anterior and posterior corneal curvature measurements. The final image represents a 3D topography that includes various colour-coded maps (curvature, elevation, pachymetry) of the entire corneal surface. It also provides data on anterior chamber depth, the corneal white-to-white diameter, and data from the anterior surface of the iris and lens. These devices are useful for diagnosis, follow-up, and management of corneal ectasia.

One needs to keep in mind that the representation of the posterior corneal surface may not be accurate as posterior elevation maps are derivatives and not the actual measurements.

## Scheimpflug imaging

Pentacam is a device that uses a rotating Scheimpflug camera to generate a 3D model of the anterior segment. It provides information, such as corneal and lens densitometry for opacification, keratometry, colour-coded maps for corneal thickness, elevation, curvature, or refractive power (or four maps refractive), pupil diameter and anterior chamber analysis. A popular feature, known as Belin-Ambrósio enhanced ectasia display (BAD) helps in detecting early cases of ectasia and is useful in screening candidates for refractive surgery.

Pentacam also helps in patients with previous refractive surgery and cataract, and in determining corneal aberrations. Galilei is a newer device that uses a dual Scheimpflug camera and incorporates Placido disc technology to improve curvature information on the central cornea.

The advantages of these devices are their accuracy, ease of use, repeatability, speed, quality and holistic anterior segment analysis.

The initial steep learning curve for data and image interpretation is a limitation for using Scheimpflug imaging. Image resolution, visualisation of iris and anterior chamber details may be better with ultra- high-resolution OCT. Pentacam's accuracy in the case of corneal scars is limited, in which case ultrasound bio-microscopy (UBM) may be a better option to visualise the anterior segment structures.

## Optical coherence tomography (OCT)

Anterior segment OCT (ASCOT) captures dynamic high-resolution cross-sectional images of the ocular surface and anterior segment in a non-invasive manner.^2^ OCT captures images with ease and interpretation of the images is not difficult. OCT is used for several investigations such as:

- Ocular surface disorder and dry eye disease: tear meniscus height and meibomian gland assessment
- Assessment of corneal opacities: endothelial gutta, depth of scarring, corneal thickness
- Keratoplasty workup and follow-up: assessment of corneal thickness and opacity, especially for lamellar/partial thickness surgeries
- Keratoconus: evaluation of focal corneal thinning and asymmetry; epithelial thickness measurement; visualisation of depth of demarcation line after collagen cross-linking; diagnosis and management of hydrops in keratoconus
- Corneal infections: assessment of depth of infiltrates, areas of necrosis, endothelial plaque
- Refractive surgery: assessment of flap thickness, interface details; workup for phakic intraocular lens for myopia
- Anterior segment tumours: ocular surface squamous neoplasia, stromal iris cysts and conjunctival nevi
- Others: corneal deposits (Kayser–Fleischer ring, drug deposits) and intracameral foreign body
- Intra-operative OCT: integration with operating microscope helps in lamellar keratoplasty and ocular surface reconstruction (Figure 2A and B)

## Confocal microscopy

In vivo confocal microscopy (IVCM) is a minimally- invasive bio-imaging technique that allows high-resolution analysis of corneal microstructure and function.^3^ IVCM is useful in:

- diagnosing and managing acanthamoeba and fungal keratitis
- detecting deep-seated infections thereby preventing corneal scraping for microbiological diagnosis diagnosing corneal dystrophies and deposits
- gaining a better understanding of dry eye disease
- studying long-term changes in corneal backscatter, corneal nerves, and cellularity

Confocal microscopy has provided more insights into visual quality after lamellar keratoplasty, excimer kerato-refractive surgery and corneal alterations after contact lens wear.

## Ultrasound biomicroscopy (UBM)

UBM is a high-frequency ultrasound used to capture images of the anterior segment. The procedure involves placing a fluid-filled eyecup over the eye and immersing the probe into the fluid to visualise the anterior segment. It allows deeper penetration and imaging through corneal opacities, dynamic view of the anterior segment structures and visualisation of the ciliary body, which, may not be possible with an OCT examination.

UBM is a contact procedure, it requires patient cooperation, and a highly-skilled operator to get good quality images which might sometimes be a challenge.

## Ocular surface analyser

Ocular surface analyser (OSA) is a new addition to the plethora of imaging devices. It helps in non-invasive analysis of tear film, enables quick and detailed structural research of the tear composition and tear film layers.^4^ It also helps to identify the type of dry eye disease and determine targeted treatment for individual layers. OSA is helpful in several investigations such as:

- interferometry- measurement of tear film stability, thickness, and pattern of the lipid layer
- tear meniscus- helps to check its height, regularity, and shape
- non-invasive break up time(NIBUT)- using grids projected onto the cornea, it measures, the stability of the mucin layer and the entire tear film
- meibography- images the shape of the meibomian gland through transillumination of the eyelid with infrared light, helps in picking up drop-out areas, and diagnosis of the meibomian gland dysfunction
- others- ocular redness classification, blink rate, pupillometry (scotopic, mesopic, and photopic)

While we have come a long way with the available investigative modalities, a thorough clinical examination is crucial for correlation and appropriate management.

**Figure 2A:**
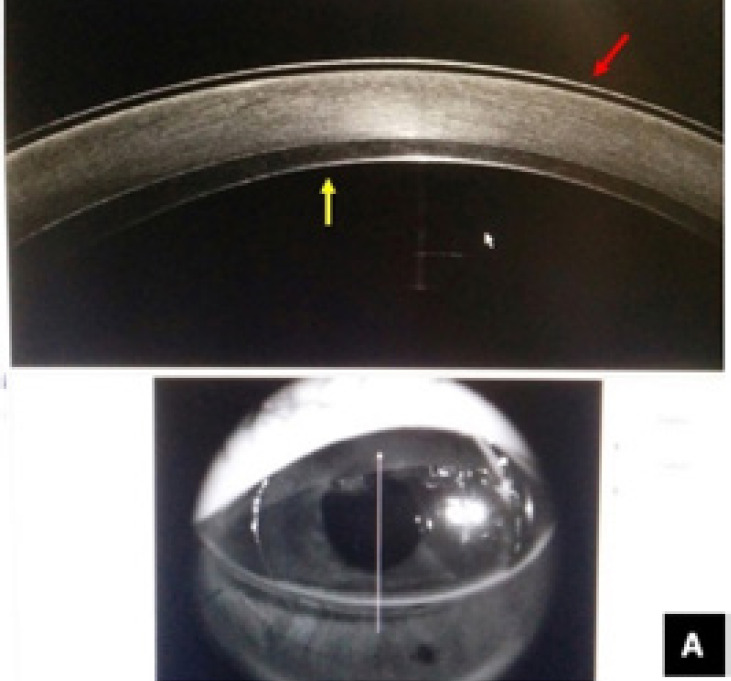
RTVue ASOCT image showing well attached DSAEK graft (yellow arrow) with a contact lens in situ (red arrow)

**Figure 2B:**
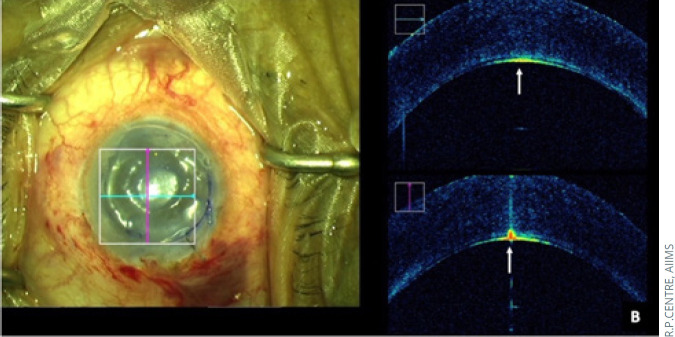
Intraoperative OCT image with a well attached DMEK graft (white arrow) at the end of surgery.
